# Construction and validation of a risk prediction model for acute kidney injury in patients after cardiac arrest

**DOI:** 10.1080/0886022X.2023.2285865

**Published:** 2023-11-23

**Authors:** Liangen Lin, Linglong Chen, Yingying Jiang, Renxian Gao, Zhang Wu, Wang Lv, Yuequn Xie

**Affiliations:** Departments of Emergency, Wenzhou People’s Hospital, Wenzhou Maternal and Child Health Care Hospital, Wenzhou, Zhejiang, China

**Keywords:** Cardiac arrest, acute kidney injury, independent risk factors, predictive model, nomogram

## Abstract

**Objective:**

Identifying patients at high risk for cardiac arrest-associated acute kidney injury (CA-AKI) helps in early preventive interventions. This study aimed to establish and validate a high-risk nomogram for CA-AKI.

**Methods:**

In this retrospective dataset, 339 patients after cardiac arrest (CA) were enrolled and randomized into a training or testing dataset. The Student’s *t*-test, non-parametric Mann-Whitney *U* test, or *χ*2 test was used to compare differences between the two groups. Optimal predictors of CA-AKI were determined using the Least Absolute Shrinkage and Selection Operator (LASSO). A nomogram was developed to predict the early onset of CA-AKI. The performance of the nomogram was assessed using metrics such as area under the curve (AUC), calibration curves, decision curve analysis (DCA), and clinical impact curve (CIC).

**Results:**

In total, 150 patients (44.2%) were diagnosed with CA-AKI. Four independent risk predictors were identified and integrated into the nomogram: chronic kidney disease, albumin level, shock, and heart rate. Receiver operating characteristic (ROC) analyses showed that the nomogram had a good discrimination performance for CA-AKI in the training dataset 0.774 (95%CI, 0.715–0.833) and testing dataset 0.763 (95%CI, 0.670–0.856). The AUC values for the two groups were calculated and compared using the Hanley-McNeil test. No statistically significant differences were observed between the groups. The calibration curve demonstrated good agreement between the predicted outcome and actual observations. Good clinical usefulness was identified using DCA and CIC.

**Conclusion:**

An easy-to-use nomogram for predicting CA-AKI was established and validated, and the prediction efficiency of the clinical model has reasonable clinical practicability.

## Introduction

Cardiac arrest (CA) is a sudden and devastating event characterized by a low survival rate and poor prognosis, and is the leading cause of cardiovascular death [1]. According to global statistics, an estimated 4.5 million lives are lost annually owing to CA, with China accounting for approximately 544,000 of these cases [2]. In China, the incidence of CA is 40.7 per 100,000 individuals, with only 4.0% of patients achieving successful recovery of spontaneous circulation (ROSC) [3]. The survival rate for out-of-hospital cardiac arrest (OHCA) is less than 1%, underscoring the gravity of this condition [2,4].

A large meta-analysis demonstrated the substantial impact of cardiac arrest-associated acute kidney injury (CA-AKI) on the early and long-term prognosis of cardiac arrest patients, with a reported prevalence of 40.3% [5]. Patients with CA-AKI exhibited higher disease severity scores, increased rates of organ failure, and heightened mortality rates [6,7]. Moreover, scholars have found that the occurrence of acute kidney injury (AKI) is significantly associated with poor neurological prognosis and increased risk of chronic kidney disease in CA patients [8,9].

Identifying AKI in its early stages is crucial for CA patients as there is currently no effective treatment other than renal replacement therapy (RRT) when the condition progresses severely. Several studies have developed risk prediction models for the identification of AKI in critically ill patients, which incorporates male, hypertension, chronic kidney disease (CKD), shock and cardiac failure, but every model has its limitations, as they fail to effectively identify and distinguish specific diseases [10–12]. Moreover, the models lack of comprehensive validation necessitates further investigation. Hence, the development of risk prediction models for CA-AKI is necessary. This will enable clinicians to promptly and accurately identify AKI, leading to timely intervention before the condition progresses to a severe stage.

## Methods

### Data source

Within the confines of our present retrospective dataset, all patients who regained ROSC were deemed eligible for screening at the Wenzhou People’s Hospital between January 2018 and December 2022. All subjects enrolled in the study were required to satisfy each of the following inclusion criteria: (1) age ≥ 18 years; (2) diagnosis of cardiac arrest, with subsequent admission to the intensive care unit (ICU) following ROSC; Subjects were excluded if they met any of the following criteria: (1) patients with missing data; (2) length of stay less than 24 h; (3) patients with end-stage renal disease who required hemodialysis or had a pre-procedural estimated glomerular filtration rate (eGFR) below 15 mL/min/1.73 m^2^; (4) trauma-induced cardiac arrest; (5) active malignant tumor on admission; and (6) patients in pregnancy or lactation.

To ensure the accurate and Transparent Reporting of research findings, this study was conducted in accordance with the Transparent Reporting of a multivariable prediction model for Individual Prognosis or Diagnosis (TRIPOD) guidelines [13], and utilize the Prediction Model Risk of Bias Assessment Tool (PROBAST) assessment tool for risk of bias evaluation [14].

### Patients and data variables

Baseline variables incorporated a variety of traditional demographic data, along with candidate predictors for CA-AKI that were identified through careful consideration of previous research, biological plausibility, and/or expert consensus derived from clinical experience. Furthermore, a predictive model was developed considering the availability of variables, thereby ensuring broad applicability across diverse clinical settings. The demographic characteristics included age, sex, hypertension, diabetes, coronary artery disease, and CKD. The hemodynamic characteristics included shock, heart rate, and mean arterial pressure (MAP). The cardiac arrest characteristics included cardiac cause, shockable rhythm, time to ROSC, and ou-of-hospital care. Upon admission, blood samples were obtained as the first available laboratory data after ROSC. These samples were processed according to the local laboratory standards. Laboratory tests included serum creatinine (Scr), albumin, and lactate levels. Patients with covariates with a prevalence of less than 5% and those with > 50% missing data were excluded.

Variables were delineated in accordance with established standard criteria. Diabetes mellitus was defined using the following criteria: fasting plasma glucose ≥7.0 mmol/L, 2-h plasma glucose ≥11.1 mmol/L during an oral glucose tolerance test, HbA1c ≥6.5%, or documented medical history of diabetes [15]. Hypertension was defined as any of the following criteria: systolic blood pressure (SBP) ≥ 140 mmHg, diastolic blood pressure (DBP) ≥ 90 mmHg, or current use of antihypertensive medications. MAP and heart rate were assessed by calculating the median value from multiple measurements obtained within a 30-min timeframe following admission to ICU. The formula for calculating MAP: MAP (mmHg) = (SBP + 2 × DBP)/3 [16]. The shock was defined as persistent hypotension, indicated by an SBP < 90 mmHg, despite adequate fluid resuscitation or administration of vasopressor agents (e.g., dopamine/dobutamine, adrenaline) for a duration exceeding 6 hours [17].

CA-AKI was the primary outcome assessed in this study. Scr levels were determined at admission and within 72 h post-admission. CA-AKI was determined based on elevated serum creatinine levels according to the diagnostic criteria of KIDGO [18]. These criteria include: (1) an increase in Scr ≥ 26.5 μmol/L within 48 h; and (2) an increase in Scr exceeding 1.5 times the baseline value, known or inferred to have occurred within 7 d.

### Development and validation of the prediction model

**Figure 1.**
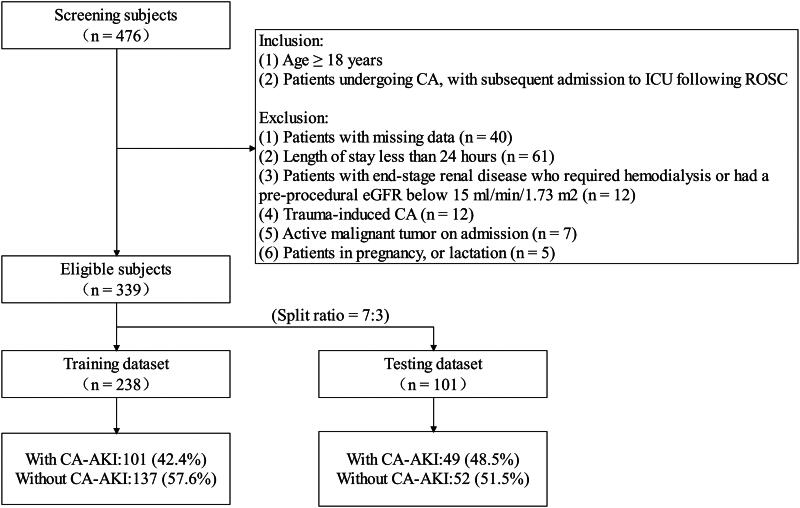
The flow chart of the current study. CA: cardiac arrest; ICU: intensive care unit; ROSC: recovery of spontaneous circulation; eGFR: estimated glomerular filtration rate. CA-AKI: cardiac arrest-associated acute kidney injury.

Upon randomization, the subjects were allocated to two distinct datasets, the training group and the testing group, with a split ratio of 7:3. The training dataset was used to establish a predictive nomogram, and the testing dataset was employed to validate its efficacy. Figure 1 shows the flowchart of patient selection. To adhere to the recommended guideline of having approximately 10 to 15 times the number of ending events as the number of independent variables in the regression equation, the training dataset underwent variable selection using the Least Absolute Shrinkage and Selection Operator (LASSO) method [19]. The LASSO algorithm employed tenfold cross-validation to estimate the weight of its penalty term, denoted as lambda (λ). The indicator *λ* is instrumental in determining the model’s complexity. Specifically, a value of *λ* = 0 has no effect on the regression parameters, while as *λ* →∞, the regression parameters tend to shrink until they eventually become 0. The one-standard-error rule to select the optimal tuning parameter *λ* (The optimal *λ* value was determined by selecting the highest *λ* for which the mean-squared error fell within one standard deviation of the minimum). Ultimately, the LASSO method identifies several non-zero estimates. This approach aimed to select significant predictors while avoiding both overfitting and underfitting of the model.

### Statistical analysis

Continuous variables were presented as median (interquartile range) and compared using the Mann-Whitney U test, while categorical variables were represented by count (proportion) and compared using the chi-square test or Fisher’s exact test. To enhance model generalizability and mitigate overfitting concerns, initial screening was performed using the LASSO technique to identify potential predictors. Subsequently, a multivariate logistic regression analysis was conducted, followed by the construction of a nomogram that incorporated all identified independent prognostic factors.

The nomogram was validated using multiple metrics for assessment. Receiver operating characteristic (ROC) curves were used to assess discrimination performance in both the training and testing datasets. The calibration of the predictive equations was assessed using methods such as a calibration slope curve, Hosmer and Leme show goodness-of-fit test, and Harrell unreliability test. The clinical usefulness of the nomogram was evaluated using decision curve analysis (DCA) and a clinical impact curve (CIC) across clinically relevant threshold probabilities. The accuracy of the risk model was assessed using measures such as net reclassification improvement (NRI) and integrated discrimination improvement (IDI) values, which also served as a baseline reference for model improvements. Statistical significance was defined as a two-sided *P*-value of <0.05. Statistical analyses were performed using the SPSS software (version 25.0; SPSS Inc., Chicago, IL, United States) and R version 3.3.2 (The R Foundation for Statistical Computing, Vienna, Austria). The utilized software packages encompassed tidyverse, mice, caret, leaps, glmnet, rms, pROC, and ggDCA.

## Results

### Baseline characteristics of the study cohort

A comprehensive screening process identified 339 patients with cardiac arrest in this dataset. The subjects were randomized into the training dataset (238 patients) and testing dataset (101 patients) according to a split ratio of 7:3. Table S1 displays patient characteristics according to the dataset. Furthermore, patients with CA were categorized into two distinct cohorts, specifically the AKI and non-AKI groups, based on the development of AKI within the initial seven days of ICU admission. The overall AKI occurrence rate was 44.2% (150/339). Further analysis revealed that stage 1, stage 2, and stage 3 AKI was observed in 17.7% (60/339), 9.4% (32/339), and 17.1% (58/339) of the cases, respectively. The difference in CA-AKI prevalence between the datasets was not significant (training vs. testing:42.4% vs. 48.5%, *p* = .303).

Table 1 shows the bivariate analyses according to CA-AKI in the training dataset. Patients with CA-AKI had a higher incidence of CKD (32.7% vs. 13.1%, *p* < .001). Additionally, CA-AKI patients had relatively poor hemodynamic parameters (lower MAP levels, higher prevalence of heart rate and shock) and abnormal laboratory indicators (lower albumin, higher Scr, and lactate) (all *p* < .05).

**Table 1.** Summary of predictive variables according to CA-AKI in the training dataset.

Predictive variables	Training dataset		*p* value
	without CA-AKI *n* = 137	CA-AKI *n* = 101	
Demographic characteristics			
Age, year	62.9 ± 17.6	61.6 ± 18.6	.571
Male, *n*(%)	94 (68.6)	67 (66.3)	.711
Hypertension, *n*(%)	53 (38.7)	45 (44.6)	.363
Diabetes, *n*(%)	29 (21.2)	23 (22.8)	.767
Coronary artery disease, *n*(%)	30 (21.9)	22 (21.8)	.983
CKD, *n*(%)	18 (13.1)	33 (32.7)	<.001
Cardiac arrest characteristics			
Non-shockable rhythm, *n*(%)	25 (18.2)	16 (15.8)	.627
Time to ROSC, min	15.0 (8.0, 29.0)	17.0 (10.0, 30.0)	.106
Out of Hospital, *n*(%)	89 (65)	61 (60.4)	.471
Non-cardiac cause, *n*(%)	31 (22.6)	19 (18.8)	.475
Hemodynamics characteristics			
MAP, mmHg	88.1 ± 20.4	79.2 ± 20.7	.001
Heart rate, beats/min	94.0 (82.0, 115.0)	110.0 (91.0, 128.0)	.001
Shock, *n*(%)	82 (59.9)	89 (88.1)	<.001
Laboratory testing			
Creatinine, μmol/L	91.0 (76.0, 110.0)	105.0 (84.0, 121.0)	.004
Lactate, mmol/L	7.5 (3.5, 10.4)	9.6 (6.0, 12.0)	<.001
Albumin, g/L	33.7 ± 6.7	30.2 ± 6.5	<.001

Data presented are mean ± SD, median (25th-75th percentile), or *N* (%).

CKD: Chronic Kidney Disease; ROSC: restoration of spontaneous circulation; ICU: intensive care unit; CA-AKI: contrast-associated acute kidney injury.

### Predictor selection and nomogram development

**Figure 2.**
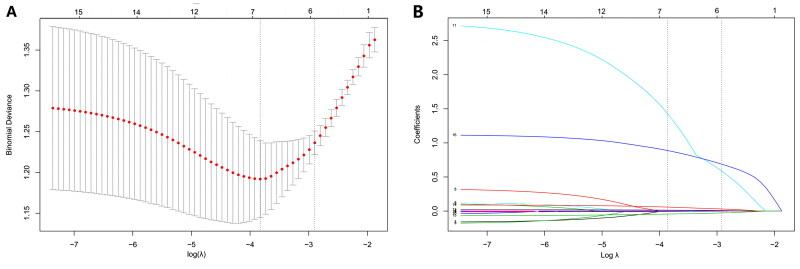
Predictive variables selection by the least absolute shrinkage and selection operator (LASSO) algorithm. (A) Identification of the optimal penalization estimate of lambda. The optimal penalization estimate of lambda was determined through tenfold cross-validation, employing a minimum error criterion in LASSO regression. (B) The LASSO estimate profile of predictive variables. The left vertical line represents the position of the optimal lambda, while the right vertical line corresponds to one standard error away from the optimal lambda.

**Figure 3.**
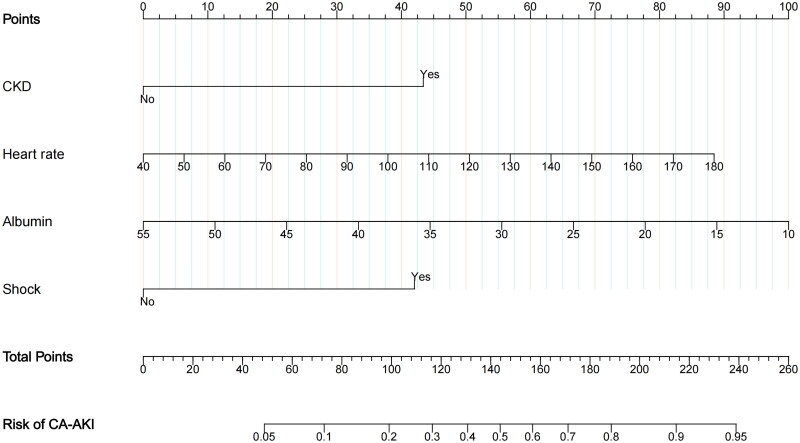
Nomogram to estimate the probability of CA-AKI. A nomogram for CA-AKI was constructed using CA data. Each predictive variable is represented by a point on the uppermost scale, and the total points are the sum of all predictors. The total points for each patient indicate the probability of developing CA-AKI. The population distribution in the training dataset can be visualized through either density plots or box sizes for each predictor.

The least absolute shrinkage and selection operator algorithm was used to determine a set of optimal predictors for CA-AKI (Figure 2A,B). Six predictive variables were screened out by LASSO regression, including CKD, albumin, shock, heart rate, MAP, and lactate. A stepwise logistic regression model was used to filter out redundant candidate variables and construct a prediction model. Eventually, four identical predictors were determined using the LASSO algorithm and logistic regression model (Table 2). Furthermore, these factors were assembled into a predictive model and visualized using a nomogram in the training dataset (Figure 3). A positive correlation between the score and risk of AKI development in CA patients suggests that higher scores are indicative of elevated risk levels.

**Table 2.** Prediction factors for AKI in cardiac arrest patients.

Variable	Univariate analysis				Multivariate analysis			
	*B*	OR	95%CI	*p* value	*B*	OR	95%CI	*p* value
Intercept	−2.049				−1.451			
CKD	0.574	3.21	1.68–6.13	<0.001	1.344	3.92	1.85–8.32	<.001
MAP	−0.002	0.98	0.97–0.99	0.001				
Heart rate	0.006	1.02	1.01–1.03	0.002	0.020	1.02	1.01–1.03	.001
Lactate	0.030	1.15	1.07–1.24	<0.001				
Albumin	−0.026	0.92	0.88–0.96	<0.001	−0.069	0.94	0.90–0.99	.014
Shock	0.682	4.97	2.49–9.95	<0.001	1.301	2.90	1.34–6.25	.007

AKI: Acute Kidney Injury; OR: Odds ratio; CI: Confidence interval; *B*: Regression coefficient; CKD: Chronic Kidney Disease; MAP: Mean Arterial Pressure.

### Evaluation of the effectiveness of the nomogram

**Figure 4.**
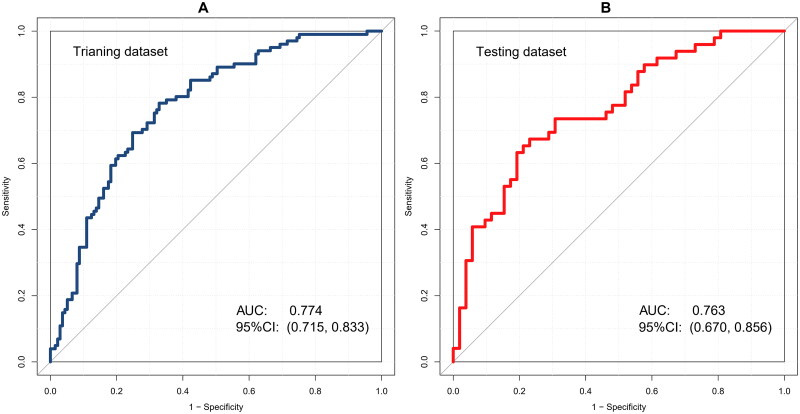
Receiver operating characteristic (ROC) analyses. Nomogram demonstrated good discrimination for CA-AKI in (A) training dataset 0.774 (95% CI, 0.715–0.833) and (B) testing dataset 0.763 (95% CI, 0.670–0.856). AUC: area under the curve; CI: confidence interval.

**Figure 5.**
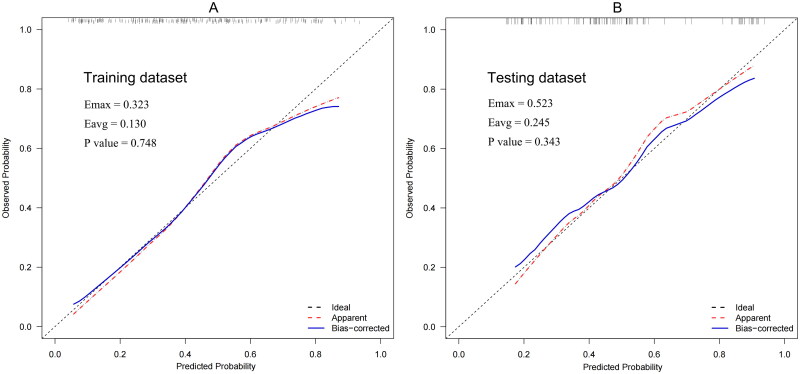
Calibration plots in training and testing dataset. The calibration plots exhibited high accuracy in predicting absolute risk, as evidenced by the result of panel (A) the training dataset (p = .748) and (B) the testing dataset (p = .343). a good calibration of the nomogram is indicated by a 45° diagonal line, reflecting the relationship between the actual rate (y-axis) and predicted probability (x-axis) of CA-AKI. p > .05 indicates a satisfactory calibration with no significant disparity between the observed rate and the predicted probabilities.

Receiver operating characteristic analyses were conducted to estimate the discrimination power of this nomogram, and the area under the curve (AUC) was computed. Furthermore, a good discrimination power was verified in the training dataset 0.774 (95%CI, 0.715–0.833) and testing dataset 0.763 (95%CI, 0.670–0.856), respectively (Figure 4A,B). AUC values were compared using the Hanley–McNeil test, and no statistically significant difference was found between the two groups (*p* = .063). The calibration of the predictive equations was assessed using various methods, including the calibration slope curve, the Hosmer–Lemeshow goodness-of-fit test, and the Harrell unreliability test. Excellent concordance was identified between the predicted and observed probabilities in the training dataset (*p* = .748) and testing dataset (*p* = .343) (Figure 5A,B).

### Nomogram validation by clinical usefulness assessment

**Figure 6.**
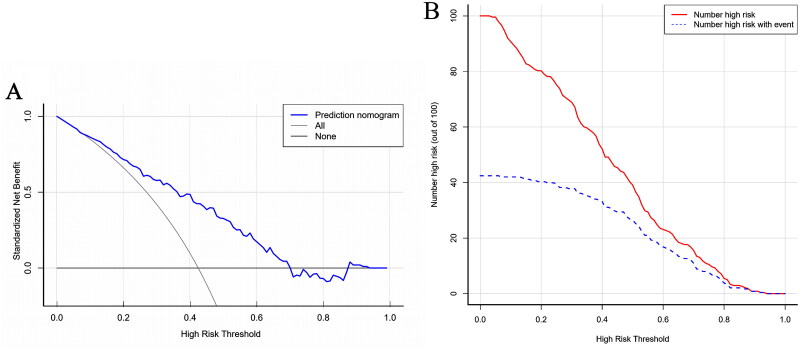
Decision curve analysis and clinical impact curve in testing in training dataset. (A) Decision curve analysis. When applying the nomogram for CA-AKI prediction, utilizing a threshold probability ranging from 3.5% to 70% yields greater benefits compared to both the treat-all-patients approach and the treat-none approach. Blue solid line indicates the nomogram; diagonal line, assumes the presence of CA-AKI in all patients; horizontal line, assumes the absence of CA-AKI in any patient. (B) Clinical impact curve. Through the utilization of the resample bootstrap method, clinical impact curve analysis predicted the probability stratification of 1000 subjects. The plot illustrates the count of high-risk patients identified by the nomogram (red solid line) and the number of high-risk patients who experienced CA-AKI occurrence (blue dashed line) for each threshold probability.

DCA and CIC were used to assess the clinical usefulness. The DCA curve suggests that the nomogram contributes to the net benefit in almost all threshold probabilities, especially in the range of 0.035–0.700 (Figure 6A) in the training dataset. When the threshold probability was <0.035, the net benefits of the nomogram were not superior in predicing CA-AKI in all patients. When the threshold probability was ≥0.700, the net benefits of the nomogram were not superior in predicting no patients suffering from CA-AKI. In the testing dataset, the nomogram demonstrated a net benefit ranging from 0.141 to 0.833 (Figure S1). The CIC of the nomogram depicted the predicted number of CA-AKI and true-positive patients at different threshold probabilities (Figure 6B). This nomogram demonstrated good clinical usefulness.

### Sensitivity analysis

Sensitivity analyses were conducted to assess the uncertainty of the prediction model. During the establishment of the predictive model, patients treated from January 2018 to December 2021 were allocated to the training dataset (255 patients), while those treated from January 2022 to December 2022 constituted the testing dataset (84 patients) for constructing the CA-AKI risk prediction model. The model was constructed using the same methodology as the prediction model.

The training dataset was subjected to the LASSO algorithm and the multivariate logistic regression model, leading to the screening of four predictors (CKD, albumin, shock, and heart rate) and the construction of predictive models. The prediction model successfully identified appropriate CA patients in both the training dataset (AUC = 0.777) and the testing dataset (AUC = 0.751) (Figure S2). Additionally, the calibration curve demonstrated a strong agreement with the observed presence of CA-AKI (Figure S3). This consistency suggests that the model exhibits good performance even for individuals from different centers.

## Discussion

In this retrospective observational dataset, using low-cost, readily available clinical variables, a well-calibrated and well-discriminated nomogram for CA-AKI was established and validated in post-CA patients. This nomogram incorporated four easily available predictors and demonstrated good clinical usefulness as verified by DCA and CIC. Its implementation enables physicians to identify high-risk patients with CA-AKI and optimize their clinical decision-making.

Acute kidney injury is a common complication after CA and can lead to a poor prognosis [7]. Certain preventive interventions, such as enhancing hemodynamic parameters, implementing a restrictive liquid management strategy, and administering vasoactive agents, have demonstrated efficacy in reducing the incidence of CA-AKI [20–22]. The implementation of intensive preventive measures has the potential to substantially benefit high-risk CA-AKI patients, highlighting the criticality of the timely identification of such individuals. Risk factors for CA-AKI reported in previous studies include older age, male sex, CKD, shock, high heart rate and lactate levels, lower MAP levels, and cardiac arrest characteristics. Nevertheless, certain studies investigating these interventions are limited in their findings owing to inadequate information on reproducibility and small sample sizes.

In the present nomogram, four easily accessible predictors, namely demographic characteristics (CKD), hemodynamic characteristics (heart rate, shock), and laboratory testing (albumin), were determined and incorporated. A correlation exists between demographic characteristics and the incidence of CA-AKI. AKI and CKD exhibit a close relationship, with CKD serving as a risk factor for AKI and contributing to the development of CKD [9,23]. Consistent with our expectations, our findings revealed a significantly higher proportion of patients with CKD in the AKI group than in the non-AKI group (32.7% vs. 13.1%), highlighting the increased prevalence of AKI among individuals with CKD. This phenomenon can be attributed to the pathophysiological mechanisms of organ ischemia-reperfusion injury [24].

Hemodynamic characteristics were also predictive of CA-AKI. Heart rate and shock have been identified as significant cardiovascular risk markers. AN increased heart rate observed during subfreezing temperatures is indicative of elevated mortality rates, unfavorable neurological outcomes, and an elevated risk of CA-AKI [25]. Our study demonstrated a significant positive correlation between heart rate and the incidence of CA-AKI. With each 10 beats/min increment in heart rate, there was a 20% corresponding increase in the risk of developing CA-AKI. Shock is an autonomous risk factor for CA-AKI [26,27]. Patients with hemodynamically stable CA exhibit an incidence of AKI at a rate lower than 10% [28]. Consistently, our study revealed a higher incidence of shock in CA-AKI patients across both training and testing groups, as opposed to the non-AKI group and was independently associated with CA-AKI. These indicators were integrated into the nomogram as risk factors for CA-AKI. According to the latest CPR guidelines, post-resuscitation hemodynamic optimization is recommended as a crucial element of post-cardiac arrest care to promote favorable neurological recovery [29]. Undoubtedly, ensuring optimal blood pressure maintenance and meticulous shock management are expected to play a pivotal role in preventing CA-AKI.

Laboratory test data could also be an underlying predictor of CA-AKI. Several observational studies have demonstrated that albumin has a protective effect against the development of CA-AKI [30–32]. However, its impact on CA patients has not been documented in the existing literature. This study revealed that a 6% reduction in the risk of CA-AKI was observed with each 1 g/L increase in the albumin level. Following ischemia-reperfusion, pronounced sympathetic nerve activity accelerates tissue catabolism and metabolism, thereby increasing oxygen demand. The activation of intracellular systems, including xanthine-xanthine oxidase, mitochondria, NADPH oxidase, and NOS, generates reactive oxygen species (ROS) that directly damage intracellular lipids and proteins, depleting adenosine triphosphate (ATP) and nutrients [33]. The subsequent release of inflammatory mediators and systemic response, along with leukocyte recruitment, exacerbates endothelial and tubular cell damage, accelerates renal function decline, and contributes to proteinuria through the involvement of the TGF-β and Wnt signaling pathways [34]. Albumin was selected using algorithms and assembled into the nomogram.

MAP and lactate levels have been established as recognized risk factors for CA-AKI [22,25]. The baseline characteristics observed in the training dataset revealed significantly lower MAP (79.2 ± 20.7 vs. 88.1 ± 20.4, *p* = .001) and elevated lactate levels (9.6 (6.0, 12.0) vs. 7.5 (3.5, 10.4), *p* < .001) in the CA-AKI group. Nevertheless, the selection algorithms did not identify MAP and lactate as definitive predictors. There may be several reasons for this finding. Potential predictors exhibiting collinearity were eliminated through LASSO regression, and as a result, heart rate and shock were chosen as independent variables in the nomogram for predicting CA-AKI. These variables may replace the predictive capacity previously attributed to MAP and lactate levels. However, when comparing the predictive models developed, which incorporated these two variables, no statistically significant disparity in predictive performance was observed (Table S2). consequently, we selected the most optimized and simplified models for clinical application.

However, this study has several limitations. First, as a retrospective review of a clinical dataset, this study is inherently influenced by the limitations commonly associated with such analyses. Second, the presence of inherent selection bias and the absence of external validation may limit the generalizability of the predictive model. Further estimation of its generalizability is warranted using additional external validation datasets. In the future, this will also be the direction we need to further study. Finally, a total of 16 screening predictors were pre-determined based on accessibility and underlying clinical significance from demographic, laboratory, and medication data, introducing the inevitable possibility of selection bias in the screening predictors. Despite these limitations, nomograms remain an accessible tool for predicting CA-AKI.

## Conclusion

In conclusion, CKD, albumin, shock and heart rate are predictors of CA-AKI. Based on the aforementioned predictors, we have developed and validated a readily applicable nomogram for predicting CA-AKI, which could serve as a noninvasive clinical tool in the clinical decision-making process. This predictive model holds promise for facilitating clinical decision-making regarding preventive interventions. Nevertheless, our predictive model only showed moderate predictive performance, thereby necessitating further enhancements in order for it to possess better clinical utility.

## Supplementary Material

Supplemental Material

Supplemental Material

Supplemental Material

Supplemental Material

Supplemental Material
