# Supplementary material for: Construction and validation of a risk prediction model for acute kidney injury in patients after cardiac arrest
Source: Ren Fail. 2023 Nov 23;45(2):2285865. doi: 10.1080/0886022X.2023.2285865 (PMC11018071; doi:10.1080/0886022X.2023.2285865)
Supplement: Supplemental Material [file IRNF_A_2285865_SM1548.pdf]

Table S2 NRI and IDI differences between the old and new models.

	Training dataset				Testing dataset			
	Estimate	Lower	Upper	*P*	Estimate	Lower	Upper	*P*
Model 1								
NRI (Categorical)	0.022	-0.048	0.092	0.54	0.019	-0.049	0.087	0.58
NRI (Continuous)	0.199	-0.057	0.454	0.13	-0.045	-0.423	0.333	0.82
IDI	0.009	-0.004	0.022	0.19	0.001	-0.003	0.005	0.55
Model 2								
NRI (Categorical)	0.019	-0.069	0.108	0.67	-0.001	-0.056	0.053	0.97
NRI (Continuous)	0.205	-0.050	0.460	0.12	-0.018	-0.408	0.372	0.93
IDI	0.012	-0.003	0.026	0.11	0.000	-0.003	0.004	0.86
Model 3								
NRI (Categorical)	-0.015	-0.117	0.088	0.78	0.019	-0.067	0.106	0.66
NRI (Continuous)	0.341	0.088	0.595	0.01	-0.020	-0.411	0.370	0.92
IDI	0.019	0.000	0.037	0.06	0.002	-0.004	0.008	0.51

Model 1: covariates included CKD, shock, heart rate, alb and MAP.

Model 2: covariates included CKD, shock, heart rate, alb and lactate.

Model 3: covariates included CKD, shock, heart rate, alb,MAP,and lactate.
