# Supplementary material for: Construction and validation of a risk prediction model for acute kidney injury in patients after cardiac arrest
Source: Ren Fail. 2023 Nov 23;45(2):2285865. doi: 10.1080/0886022X.2023.2285865 (PMC11018071; doi:10.1080/0886022X.2023.2285865)
Supplement: Supplemental Material [file IRNF_A_2285865_SM1546.pdf]

TableS1.Summary of predictive variables according to the dataset

Predictive variables	Overall<br>n = 339	Training dataset<br>n = 238	Teating dataset<br>n = 101	*P*<br>value
**Demographic characteristics**				
Age, year	62.1 ± 17.7	62.4 ± 18.0	61.4 ± 17.2	0.652
Male, n(%)	221 (65.2)	161 (67.6)	60 (59.4)	0.145
Hypertension, n(%)	142 (41.9)	98 (41.2)	44 (43.6)	0.684
Diabetes, n(%)	74 (21.8)	52 (21.8)	22 (21.8)	0.989
Coronary artery disease, n(%)	77 (22.7)	52 (21.8)	25 (24.8)	0.559
CKD, n(%)	73 (21.5)	51 (21.4)	22 (21.8)	0.942
**Arrest characteristics**				
Non-shockable rhythm, n(%)	285 (84.1)	197 (82.8)	88 (87.1)	0.316
Time to ROSC,min	15.0(9.0, 28.0)	15.0(8.0, 22.0)	15.5(9.2, 28.8)	0.265
Out of Hospital, n(%)	211 (62.2)	150 (63)	61 (60.4)	0.648
Non-cardiac cause, n(%)	271 (79.9)	188 (79)	83 (82.2)	0.503
**During ICU stay**				
MAP,mmHg	84.4 ± 19.9	84.3 ± 20.9	84.6 ± 17.5	0.891
Heart rate,beats/min	102.0 (86.0, 122.0)	101.0 (85.0, 121.0)	103.0 (86.0, 123.0)	0.665
Shock, n(%)	241 (71.1)	171 (71.8)	70 (69.3)	0.637
**Laboratory testing**				
Creatinine,μmol/L	95.0 (76.0, 114.0)	97.0 (78.0, 114.0)	88.0 (69.0, 113.0)	0.067
Lactate,mmol/L	8.1 (4.3, 12.0)	8.1 (4.6, 12.0)	7.6 (3.9, 12.0)	0.734
Albumin,g/L	31.8 ± 7.1	32.2 ± 6.8	30.9 ± 7.7	0.125
**Primary outcome**				
CA-AKI, n(%)	150 (44.2)	101 (42.4)	49 (48.5)	0.303
CA-AKI stage, n(%)				0.270
AKI stage 1	60 (17.7)	36 (15.1)	24 (23.8)	
AKI stage 2	32 (9.4)	22 (9.2)	10 (9.9)	
AKI stage 3	58 (17.1)	43 (18.1)	15 (14.9)	

Notes：data presented are mean± SD, median (25th-75th percentile), or N (%).

Abbreviations: CKD,Chronic Kidney Disease; ROSC,restoration of spontaneous circulation; ICU,intensive care unit; CA-AKI,contrast-associated acute kidney injury.

Information Classification: General
