# Supplementary figures and images for: Construction and validation of a risk prediction model for acute kidney injury in patients after cardiac arrest
Source: Ren Fail. 2023 Nov 23;45(2):2285865. doi: 10.1080/0886022X.2023.2285865 (PMC11018071; doi:10.1080/0886022X.2023.2285865)

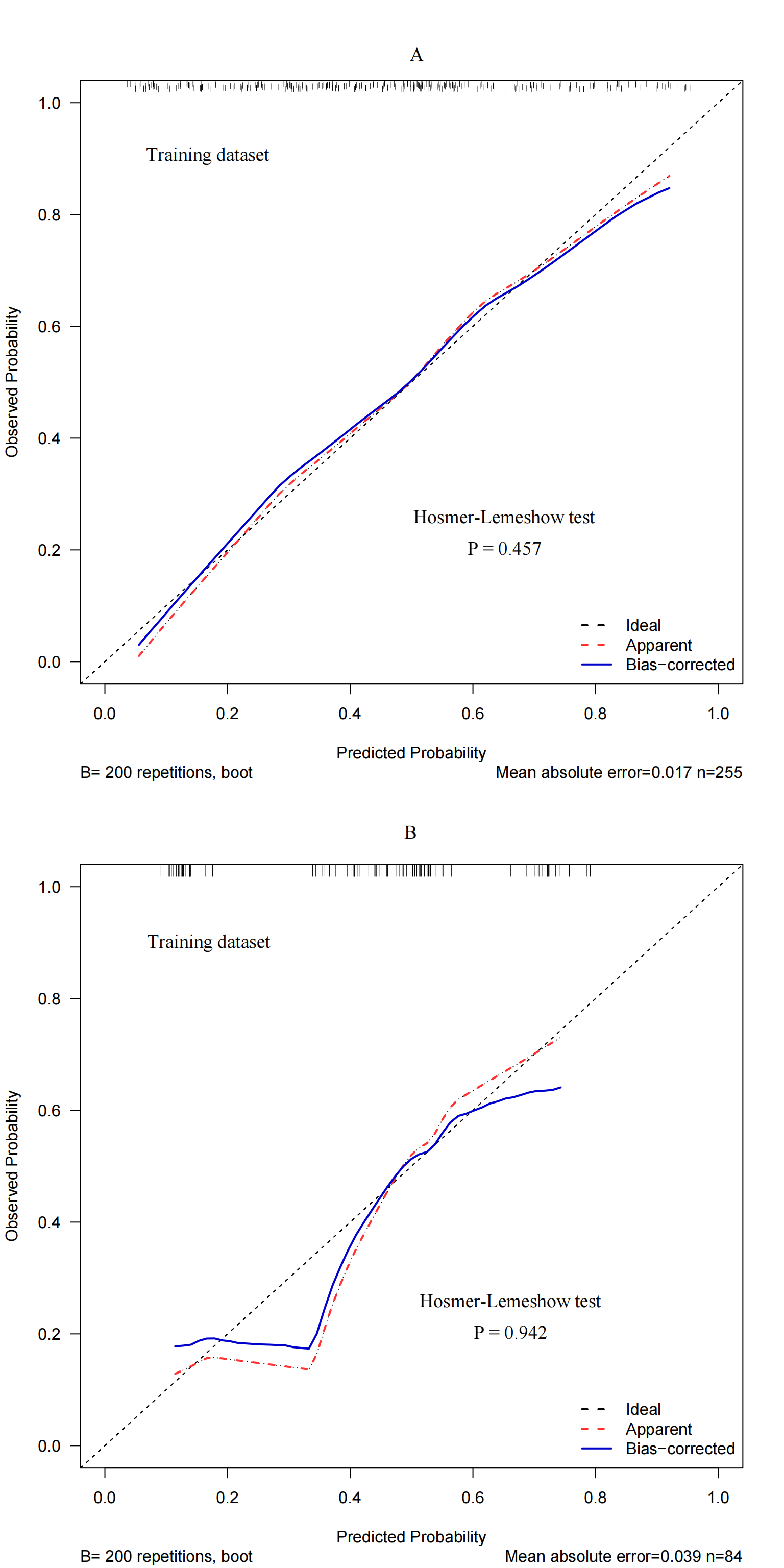

Supplement: Supplemental Material [file IRNF_A_2285865_SM1543.tif]

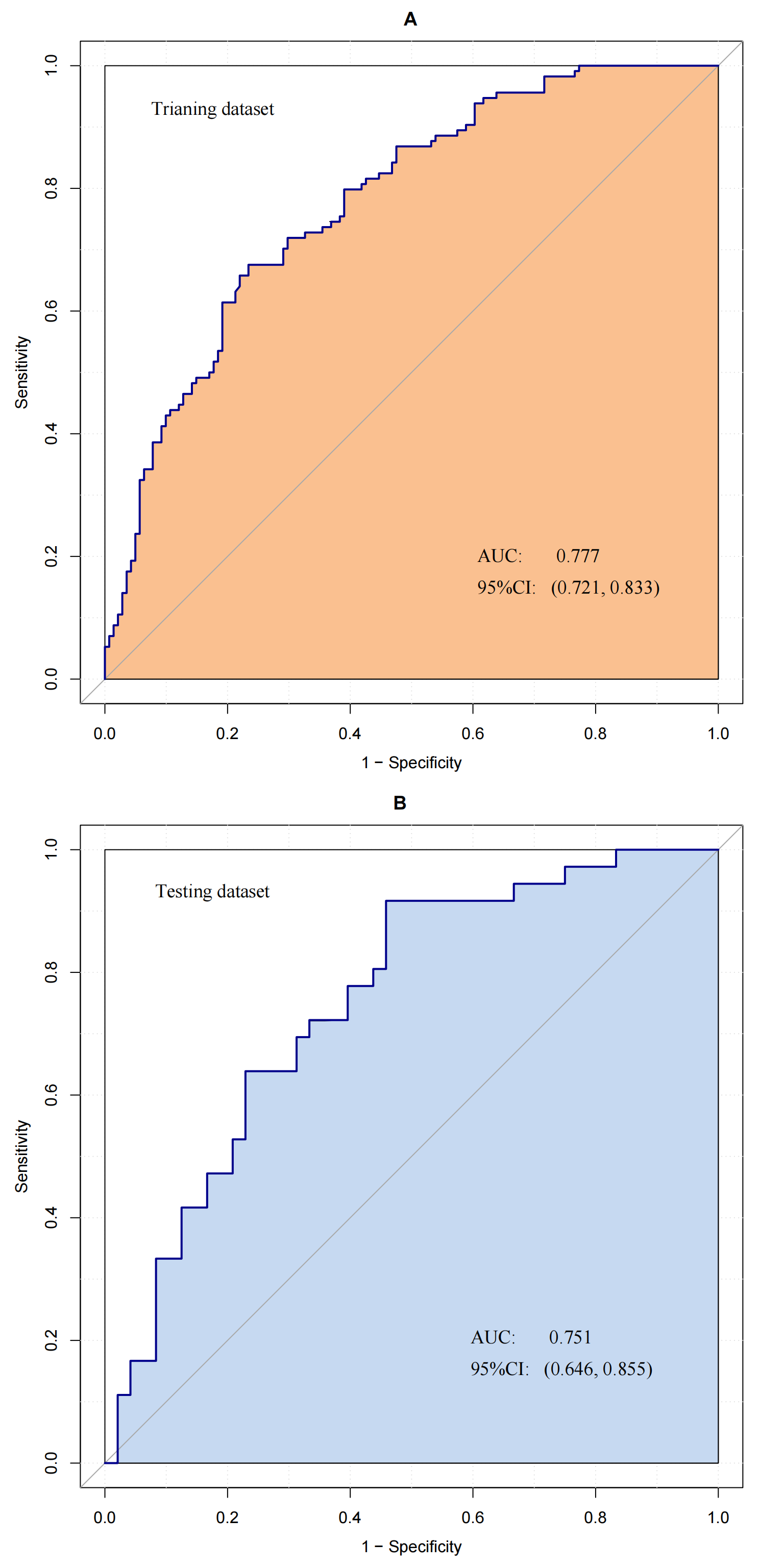

Supplement: Supplemental Material [file IRNF_A_2285865_SM1537.tif]

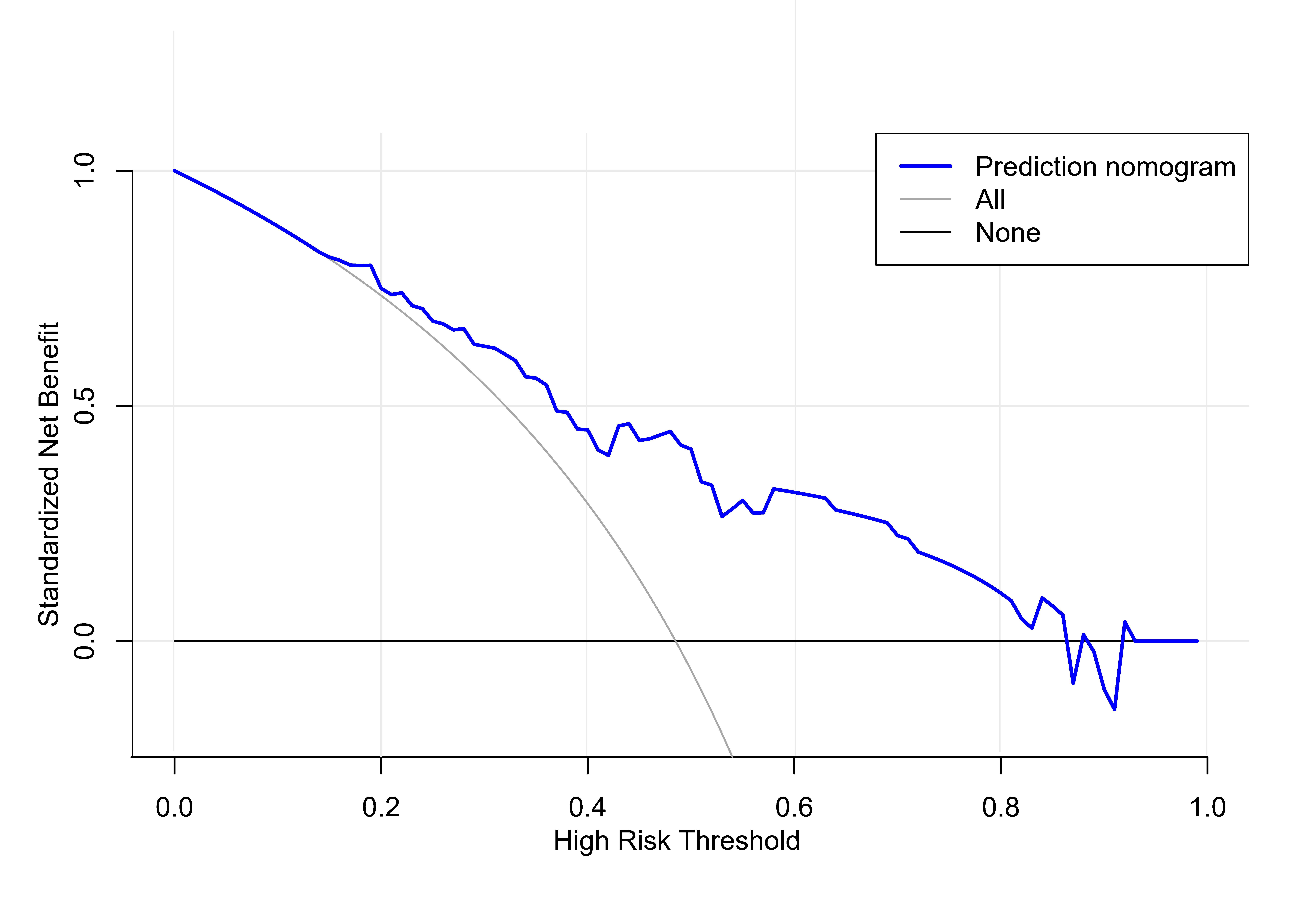

Supplement: Supplemental Material [file IRNF_A_2285865_SM1529.tiff]
